# Diversity and Distribution of Carotenogenic Algae in Europe: A Review

**DOI:** 10.3390/md21020108

**Published:** 2023-02-01

**Authors:** Konstantin Chekanov

**Affiliations:** Independent Researcher, Arabkir, 1, Yerevan 0054, Armenia; chekanov@mail.bio.msu.ru

**Keywords:** biodiversity, microalgae, carotenoids, astaxanthin, β-carotene, echinenone, adonixanthin, adonirubin

## Abstract

Microalgae are the richest source of natural carotenoids, which are valuable pigments with a high share of benefits. Often, carotenoid-producing algae inhabit specific biotopes with unfavorable or even extremal conditions. Such biotopes, including alpine snow fields and hypersaline ponds, are widely distributed in Europe. They can serve as a source of new strains for biotechnology. The number of algal species used for obtaining these compounds on an industrial scale is limited. The data on them are poor. Moreover, some of them have been reported in non-English local scientific articles and theses. This review aims to summarize existing data on microalgal species, which are known as potential carotenoid producers in biotechnology. These include *Haematococcus* and *Dunaliella*, both well-known to the scientific community, as well as less-elucidated representatives. Their distribution will be covered throughout Europe: from the Greek Mediterranean coast in the south to the snow valleys in Norway in the north, and from the ponds in Amieiro (Portugal) in the west to the saline lakes and mountains in Crimea (Ukraine) in the east. A wide spectrum of algal secondary carotenoids is reviewed: β-carotene, astaxanthin, canthaxanthin, echinenone, adonixanthin, and adonirubin. For convenience, the main concepts of biology of carotenoid-producing algae are briefly explained.

## 1. Introduction

Carotenoids are biological pigments from the tetraterpene group. They play a very important role in our life. These compounds promote a favorable course of the disease and are vital for normal functioning of the organism. They are antioxidants and photoprotectants. Some of them are precursors of vitamin A [1,2,3,4,5,6,7,8,9,10,11]. In this regard, they are components of a wide range of drugs, cosmetics, personal skincare and functional food [2,3,8,11,12,13,14,15,16,17]. Carotenoids are used as lipophilic food colorants to give a yellow, orange, or red color [14,18]. Moreover, they determine the range of colors of the amazing imagination of the observer, from blue to red, in the kingdom of animals: in crustaceans, cnidarians, mollusks, vertebrates, etc. [8,19,20,21,22,23]. However, animals cannot synthesize carotenoids de novo. Hence, these pigments should be presented in their rations. Carotenoid-containing feeds are needed in fish and poultry farms as well as in zoos.

The global carotenoid market was estimated at USD 2.00 billion in 2022; its increase to USD 2.7 billion by 2027 has been forecast [24]. Its main driving forces are the increase in the production of nutraceuticals and the growing demand for natural skincare cosmetics [25]. Europe is the largest market of carotenoids at present. This is mainly because of the well-developed animal-feeding and cosmetics sectors [25,26]. Many key players of the carotenoid world market are located in Europe: Allied Biotech Corporation (Germany), BASF (Germany), Chr. Hansen Holdings A/S (Denmark), DOHLER GmbH (Germany), DSM (The Netherlands), Dynadis SARL (France), Vidya Europe SAS (France) [25,26]. Building a climate-neutral, green, fair and social Europe is one of the main priorities of an agenda for the EU for the next five years formulated by the European Council [27]. In accordance with this plan, growth of the carotenoid industry contributes to the development of sustainable green technologies. Chemical synthesis is, so far, the main method for producing carotenoids. However, synthetic carotenoids do not have the same beneficial properties due to the difference in isomeric composition. [8,12,13,16]. For example, β-carotene, which consists of *all*-trans isomer only, does not have the same beneficial properties as the natural pigment [8] (which is the mixture of *15-cis*, *9-cis* and *all*-trans forms [9,12]). Synthetic astaxanthin, which is a mixture of three stereoisomers (*3R/3′R*, *3R/3′S* and *3S/ 3′S*), is inferior in performance to the algal pigment [11,16]. In recent years, consumers have also preferred to use products with natural carotenoids from sustainable sources [8].

Unicellular algae (microalgae) are the richest source of carotenoids [8,11,16,28,29]. Some of them can accumulate a very high amount of these pigments. This phenomenon is called carotenoigenesis. Algae are cultured on an industrial scale for carotenoid production. Two main species of microalgae are used by biotechnological manufactures: *Haematococcus lacustris* (Girod-Chantrans) Rostafinski and *Dunaliella salina* (Dunal) Teodoresco (Chlorophyta). *Haematococcus lacustris* produces red-colored ketocarotenoid astaxanthin. *Dunaliella salina* is a producer of orange β-carotene [1,8,9,12,19]. These pigments are “leaders” of the market of carotenoids [24,25]. However, there are many less elucidated species of carotenoid-producing microalgae which could be considered as new sources of these valuable biological pigments. The selection of such species is an actual task of biotechnology [8]. Many strains have been isolated in the European region. The review aims to summarize the data on diversity and distribution of carotenogenic microalgae in Europe, including continental countries, United Kingdom, Ireland, and Iceland. To cover existing data on distribution of carotenogenic algae, scientific publications (articles and chapters in books) were searched via Google Scholar with the following search parameters: algal species names and (“carotenoids” ∨ “secondary carotenoids” ∨ “Europe”). In some cases, theses and patents were also considered. Only species that had been reported as accumulating secondary carotenoids, were considered. Species names and some data on distribution were taken from AlgaeBase [30]. For convenience, the main concepts and terms of biology of carotenoid-producing algae are provided.

## 2. A Historical Note

**Figure 1:**
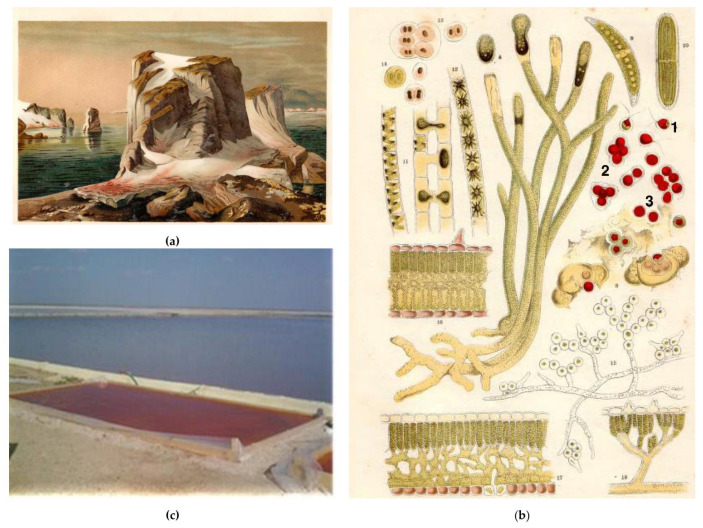
(**a**) Red snow in the Baffin Bay (illustration, XIX century, reprinted from [33] © Verlag des Bibliographischen Instituts, 1888); (**b**) stages of the life cycle of algae from snow and water, 1—motile zoospores, 2—coccoid non-motile cells, 3—reproduction by sporangia (illustration, XIX century, reprinted from [33] © Verlag des Bibliographischen Instituts, 1888); (**c**) mass cultivation of *Dunaliella salina* in PK “Galit,” Saki, Crimea, Ukraine (reprinted with permission from [37] © Springer Nature B.V., 2020).

Blood-red round spots on the snow can be seen on the snowy alpine areas on a bright sunny day in spring or autumn. At the same times of the year, so-called ‘blood rain’ can be observed. Blood rain and red (or watermelon) snow have been well-known for a long time. The earliest notes of blood red water are referred to the Bible: Second Book of Kings 3:22 [10]. Blood rain was mentioned in ancient texts by Plutarch and Cicero [31]. Aristotle first described watermelon snow in “History of Animals” [31]. Blood rain was mentioned in medieval sources, in particular, in 582 in Paris and in the IV century in Germany and North Italy [32]. It was perceived as an omen of the Black Death, the Plague epidemic in 1348–1349, that claimed the lives of many people [32]. These rains varied by their duration and intensity of coloration [32]. Nicolas-Claude Fabri de Peiresc, a French astronomer and antiquarian, hypothesized that blood rains were caused by insects. Although this hypothesis was wrong, it was the first scientific explanation of this phenomenon. Watermelon snow was described in the Savoy Mountains, in the Alps, the Pyrenees, the Carpathians, the northeastern part of the Ural Mountains, and in polar Scandinavia [33]. In 1818, John Ross described red snow in the Baffin Bay (Figure 1a) and mentioned that it appeared under bright sunlight [33]. It was established in the XIX century that watermelon snow contains red immotile unicellular organisms. Their chlorophyll was masked by a “red substance”. During snow melting they acquired flagella, became motile and started their reproduction (Figure 1b) [33].

The techniques of cultivation of red-colored photosynthetic organisms were developed in the XIX century [33]. After observation of the cultures in laboratory, it was established that green motile cells and red resting cells were related to the same species. The organism from the red snow was defined as *Sphaerella nivalis* (Baeur) Sommerfelt. The organism from small freshwater ponds with red water was called *Sphaerella pluvialis* (Flotow) Wittrock. Then, *Sphaerella nivalis* and *Sphaerella pluvialis* were transferred to the species *Haematococcus lacustris* and *Chloromonas nivalis* (Bauer) Wille., respectively [34]. Before the 1930s, the chemical nature of algal “red substance”, termed haematochrome, had not been described. The German chemist Richard J. Kuhn, a future Nobel laureate, and colleagues studied the structure of polyene pigments. They extracted and purified many carotenoids, including astaxanthin from the shells of the lobster (*Astacus gammarus*) [35]. Only in 1944 was it shown that haematochrome from algae was the same substance as astaxanthin from lobsters [19,20]. Droop determined the conditions for the accumulation of astaxanthin in microalgae: bright light and nutrient-deficient [36].

The first mentions of carotenoid-producing *Dunaliella salina* are from the XIX century. In the 1830s, Turpin noted the reddish color of water in saline lakes as *Globularia kermesia* Turpin [10]. At the same time, Dunal mentioned this same color in a salt pond on the Mediterranean coast of France due to *Haematococcus salinus* Dunal [1]. Since that time, the taxonomy of this species has undergone a series of revisions. Finally, in 1905, Teodoresco proposed the name *Dunaliella salina* [1,10]. It was mentioned in Odesa (Ukraine), Crimea (Ukraine) and Lorraine (France) [1], as well as in saline and evaporated ponds of Africa, Asia, North and South America, Australia and Antarctica [10]. 

*Dunaliella salina* is cultured for carotenoid production on an industrial scale in different countries of the world [1,9,10]. It has been recognized as a β-carotene-producing microalga since the 1960s [9]. For the first time, it was proposed as a source of carotenoids in Ukraine. In particular, pilot experiments on their large-scale culturing were carried out in Crimea [38,39] in open ponds with saline water (Figure 1c).

Nowadays, *Haematococcus lacustris* and *Dunaliella salina* are widely studied as natural sources of carotenoids. However, diversity of carotenoid producing algae is not restricted by these two species. Study of new potential producers is one the major directions of current biotechnology.

## 3. Main Concepts and Definitions of the Biology of Carotenogenic Microalgae

### 3.1. The Difference between Primary and Secondary Carotenoids

It is reasonable to divide carotenoids into two groups, i.e., primary and secondary carotenoids. Primary (or photosynthetic) carotenoids are structurally and functionally connected with photosynthetic apparatus (PSA) [28,40,41,42,43]. In PSA pigment–protein complexes containing carotenoids, a strict stoichiometry should be observed between the different components. Hence, the content of primary carotenoids is dictated by the metabolic demands of the cells and cannot reach high values [40,42,43,44,45,46]. Secondary carotenoids are not connected with PSA. Their content is not limited by stoichiometry with other cell compounds. Therefore, they can be accumulated in high quantities. The microalgae accumulating them are called carotenogenic algae [28,43,46,47,48,49].

### 3.2. Where Do Microalgae Store Secondary Carotenoids?

**Figure 2:**
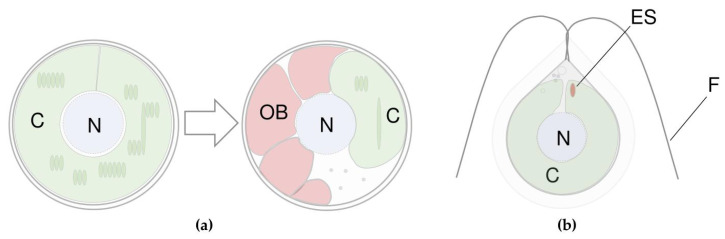
(**a**) Accumulation of astaxanthin in vegetative cells of *Haematococcus lacustris*; (**b**) zoospore of *Haematococcus lacustris*. C—chloroplast, ES—eyespot, F—flagellum, N—nucleus, OB—oil body.

A well-developed non-polar compartment is required to deposit hydrophobic molecules of carotenoids or their esters with fatty acids [50] or acylated glycosides [51]. Spherical vegetative *Haematococcus* cells do not contain significant amounts of secondary carotenoids (Figure 2a). Almost their whole volume is filled with chloroplasts. After the induction of secondary carotenogenesis, the size of PSA reduces; massive oil bodies appear in the cytoplasm. The latter serve as a storage compartment for the accumulated astaxanthin [43,46,47,52,53]. Astaxanthin accumulation is accompanied by metabolic decline and transition to the resting state, called aplanospore or haematocyst [52]. In a similar manner, *Dunaliella* accumulates β-carotene, but it takes place in chloroplast plastoglobules [54].

Asexual reproduction of *Haematococcus lacustris* could be realized through motile zoospores (Figure 1b and Figure 2b) [47]. It should be noted that zoospores exhibit stigma (eyespot) localized in the chloroplast on the apical end of the cells (Figure 2b). Notably, *Haematococcus lacustris* is one of the first objects on which the role of carotenoids in algal photoreception was studied [55]. The main components of the eyespot are eyespot globules. They differ from plastoglobules. Eyespot globules are associated with thylakoids and organized in a certain order. They contain β-carotene as a predominant pigment [56]. It is not a secondary pigment because its content is limited in the cell and because these globules are associated with PSA [56].

### 3.3. Which Carotenoids Do Microalgae Accumulate?

**Figure 3:**
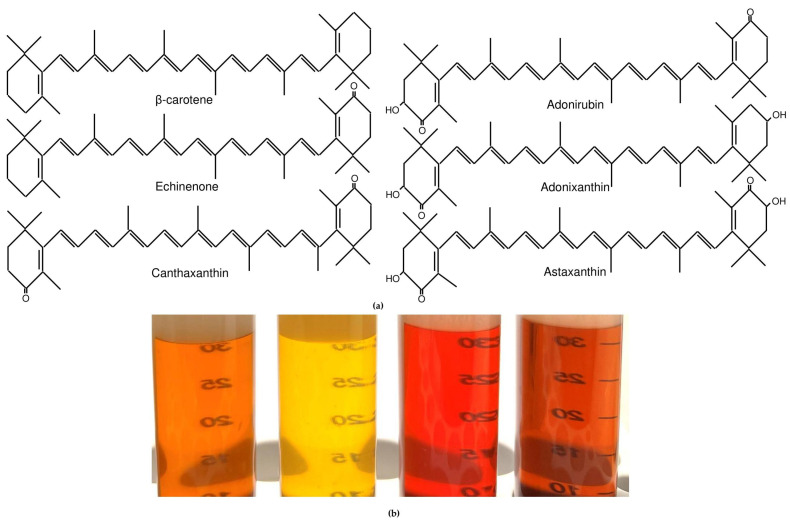
(**a**) Main secondary carotenoids of carotenogenic microalgae; (**b**) chloroform extracts of carotenogenic microalgae after induction of carotenoid accumulation (from left to right: *Deasonia granata*, *Pseudospongiococcum protococcoides*, *Haematococcus rubicundus*, *Coelastrella aeroterrestrica*).

Based on molecular structure, carotenoids are divided into oxygen-free carotenes (e.g., β-carotene) and oxygen-containing xanthophylls (e.g., astaxanthin) (Figure 3a). Ketocarotenoids are special types of xanthophylls exhibiting at least one keto group in the ionone rings. In green microalgae, it is the keto group at the fourth position of the ionone rings [42,45,46].

Based on the reported data, it seems that all eukaryotic algae accumulate either β-carotene or ketocarotenoids, derived from it, as secondary (Figure 3a) [8,11,16,28,45,46]. The spectrum of ketocarotenoids includes astaxanthin (with two hydroxyl groups and two ketogroups) as well as its precursors with 1-2 keto- and 0-2 hydroxyl groups: echinenone, canthaxanthin, adonixanthin and adonirubin (Figure 3a). Algae accumulate either one predominant or a mixture of carotenoids. *Botryococcus braunii* Kützing (Chlorophyta) also synthesize tetramethylsqualene-conjugated braunixanthines and botryoxanthines which can be considered as secondary [57,58]; however, patterns of their localization are not elucidated. Moreover, they have not been sought for practical use; thus, they will not be considered here. The composition of secondary carotenoids in microalgae dictates the color of their cells. The same is true for pigment extracts (Figure 3b).

It should be noted that although in some articles, microalgae are proposed as a source of lutein, e.g., [29,59,60,61,62,63,64,65], no reported microalgae could be considered as lutein-accumulating. Although the biomass of some algae (e.g., *Scenedesmus, Chlorella*, *Coccomyxa*, *Parachlorella*, and *Tetraselmis*) can be enriched by lutein, in all published studies, lutein content correlates with the content of chlorophylls. It means that it is a primary carotenoid. Indeed, lutein is a component of pigment–protein complexes of PSA [40,41,44] and cannot be accumulated in infinitely large amounts. The accumulation of lutein as a secondary carotenoid has been observed, for example, in petals of plants from the *Tagetes* genus, i.e., *Tagetes patula* L. and *Tagetes erecta* L. (Asteraceae). They accumulate 0.17–5.70 mg pigment per g of dry petal mass [66,67]. There, it is esterified similar to the secondary xanthophylls of microalgae [66]. The presence of lutein esterases has not been shown in microalgae.

### 3.4. Why Do Microalgae Accumulate Secondary Carotenoids?

The accumulation of secondary carotenoids in algae is a reaction to different stress factors: bright light, nutrient insufficience and the generation of reactive oxygen species (ROS) in the medium, osmotic stress, etc. It is reasonable to assume carotenogenesis as a protective mechanism against adverse environmental conditions [36,43,46,47,52]. Although secondary carotenoids are powerful antioxidants, their antioxidant properties have not been shown in algal cells [43,46,68]. Indeed, secondary carotenoids cannot be involved in the scavenging of ROS, because the main site of their formation is PSA, whereas secondary carotenoids are not associated with it. Additionally, secondary pigments are not involved in the epoxidation/de-epoxidation cycles as primary ones. Most likely, secondary carotenoids play the role of light filters (sunscreens) decreasing the amount of excessive energy absorbed by PSA. It should decrease the risk of photodamage [43,68]. Attenuation of the light absorbed by PSA in the presence of secondary carotenoids has been shown [69,70]. Indeed, they decrease the level of photodestruction under stress [68]. In addition, the synthesis of carotenoids requires oxidation in the presence of plastid terminal oxidase and plastoquinone. This process activates an alternative electron transport to decrease overreduction in the plastid electron transport chain [43].

Understanding the physiological role of secondary carotenogenesis in algae is important to explain their distribution. It is an effective protective mechanism, enabling them to dwell in habitats with adverse or even extreme conditions where all vital activity should be considered as fading [71,72,73,74,75,76,77]. 

### 3.5. Where Do Carotenogenic Microalgae Live?

**Figure 4:**
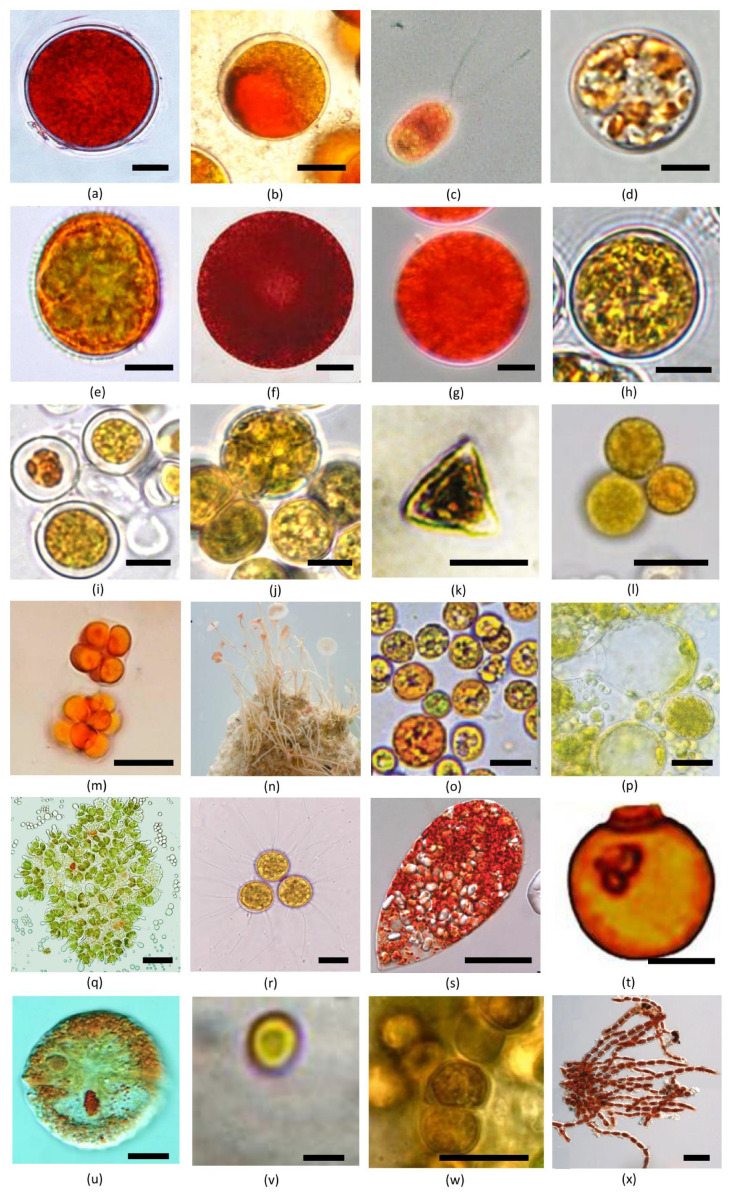
Representatives of carotenogenic algae inhabiting Europe. (**a**) *Haematococcus lacustris*, scale bar = 10 μm, adapted with permission from [69] © Springer Nature B.V., 2020; (**b**) *Ettlia carotinosa*, scale bar = 5 μm (unpublished); (**c**) *Dunaliella salina*, scale bar—not provided, adapted from [1] © Springer Nature B.V., 2005; (**d**) *Chromochloris zofingiensis*, scale bar = 5 μm, adapted from [83]; (**e**) *Chloromonas krienitzii*, scale bar = 10 μm, adapted from [84]; (**f**) *Chlainomonas* sp., scale bar = 10 μm, adapted from [85] © Taylor & Francis Online, 2018; (**g**) *Sanguina nivaloides*, scale bar = 5 μm, adapted from [86] © Oxford Academic, 2019; (**h**) *Coelastrella rubescens*, scale, bar = 10 μm, adapted from [70]; (**i**) *Bracteacoccus aggregatus*, scale bar = 10 μm, adapted with permission from [87] © Oxford Academic, 2020; (**j**) *Halochlorella rubescens*, scale bar = 5 μm, adapted with permission from [88] © Pleiades Publishing, Ltd., 2022; (**k**) *Tetraëdron minimum*, scale bar = 20 μm, adapted from [89] © Iheringia, Série Botânica, 2016; (**l**) *Deasonia* sp., scale bar = 5 μm, adapted with permission from [90]; (**m**) *Chlorosarcinopsis bastropiensis*, scale bar = 10 μm, adapted with permission from [91]; (**n**) *Acetabularia acetabulum*, scale bar—not provided, adapted from [92] © Inter-Research Science Publisher, 2012; (**o**) *Pseudospongiococcum protococcoides*, scale bar = 10 μm, adapted from [93] © Phytocenter, 2013; (**p**) *Protosiphon botryoides*, scale bar = 20 μm, adapted with permission from [94] © Taylor & Francis Online, 2019; (**q**) *Botryococcus braunii*, scale bar = 40 μm, adapted from [95]; (**r**) *Golenkinia brevispicula*, scale bar = 10 μm, adapted with permission from [96] © Elsevier, 2020; (**s**) *Euglena sanguinea*, scale bar = 20 μm, adapted with permission from [97] © Taylor & Francis Online, 2021; (**t**) *Trachelomonas volvocina*, scale bar 10 μm, adapted from [98] © TÜBİTAK libraries, 2013; (**u**) *Tovellia sanguinea*, scale bar = 10 μm, adapted with permission from [99] © Taylor & Francis Online, 2019; (**v**) *Diacronema vlkianum*, scale bar = 5 μm, adapted with permission from [100] © Elsevier, 2013; (**w**) *Rhexinema sarcinoideum*, scale bar = 10 μm, adapted from [101] © Sciendo, 2007; (**x**) *Trentepohlia jolithus* var. *yajiagengensis*, scale bar = 100 μm, adapted from [102] © PLoS, 2012.

Many species of carotenogenic microalgae are known as aeroterrestrial, i.e., they live on the border of a solid phase and air. Such an environment is characterized by a combination of adverse abiotic factors. High levels of solar radiation, sharp temperature changes and extreme water regime (frequent drying and temporal hydration in the rain) are among them [78]. These algae can be found as a reddish or orange plaque on the surface of stones, tree bark or human buildings [78,79]. They are typical for biological soil crusts (assemblages of living organisms on soil or rock surfaces in arid and semiarid areas) [74]. For example, such crusts were studied in the Alps [74]. Carotenogenic algae are common in small temporary drying ponds, such as shallow cavities of rock baths on the coasts of the seas, ponds and ornamental bird baths. There, they can form highly organized biofilms with other microorganisms [80]. To survive under harsh environmental factors, aeroterrestrial algae have effective protective mechanisms, such as ROS-neutralizing enzymes, an ascorbate–glutathione cycle, non-photochemical quenching of the excited chlorophyll states and systems of reparation of DNA and other cell components [74,78,81,82]. Speaking about their protective mechanisms, the separately ultrastructural rearrangements of the cells should be noted as a result of autophagy. This process ensured the utilization of damaged cell components and reduction in the size of PSA, which is a main site of photodamage [82]. By definition all listed mechanisms are aimed at eliminating damage; hence, they work when ROS generation and photodamage have occurred. By contrast, shielding by sunscreens, such as carotenoids, is aimed to prevent light absorption and subsequent destructive processes [81]. Accumulation of carotenoids leads to pronounced orange or red coloration of algal cells (Figure 4).

Snow algae inhabit a unique niche, for liquid water between crystals of ice. Their motile stage moves to the microenvironments with temperature and illumination optimal for growth [75,76,77,103,104]. However, for a significant portion of the time these algae exist as an immotile cyst, also known as “hypnospore” [105,106,107] resisting the adverse environmental factors. In some species, transition to the cyst is accompanied by carotenoid accumulation. Snow algae are characterized by high photostability [103]. This resistance is explained by high contents of carotenoids and α-tocopherol, as well as phenolic compounds [107,108]. They are distributed in alpine and polar glacial areas, such as the Alps, Vitosha Mountains, High Tatra Mountains, and Sierra Nevada Mountains. These algae cause red snow. In some cases, this snow also can have green, gray, brownish, orange or pink color [73]. This phenomenon plays a notable ecological role. Snow algae decrease the albedo of snow and ice surfaces, promoting ice melting in polar and alpine regions [31,73]. Snow and ice algae could be a promising object of biotechnology, because of their ability to grow at low temperatures and produce a spectrum of valuable compounds [73]. However, the low number of deposited strains is a significant disincentive to use them in biotechnological studies.

Some carotenogenic species inhabit ponds with extreme salt concentrations (up to 6 M NaCl). They accumulate protective compounds to resist external osmotic pressure. These algae cause intensive reddish or orange coloration of saline water [1,9,10,54]. These ponds are present in some European countries, e.g., salt ponds of Monzon (Spain), Marele Lacul Sărat (Romania), Sasyk and Saki lakes of Crimea (Ukraine).

## 4. Diversity and Distribution of Unicellular Carotenogenic Algae

### 4.1. Haematococcus Flotow (Chlorophyceae, Chlamydomonadales)

As was specified above, *Haematococcus lacustris* (Figure 4a) is one of the most known carotenogenic algae. It is also published under the name *H. pluvialis* Flotow, but in 2016 Nakada and Ota [109] showed in a taxonomic study, *H. lacustris* is a more correct. There are two other recently described European species of *Haematococcus*: *H. rubens* Allewaert and Vanormelingen, and *H. rubicundus* Allewaert and Vanormelingen [110]. Astaxanthin is a predominant carotenoid of *Haematococcus* spp. (c.a. 99% of total pigment content) (Table 1). It is deposited in the form of mono- and diesters with fatty acids with predominant monoesters [47,111].

The biogeography of *Haematococcus lacustris* is widely studied. It was mentioned in different countries. Most of this information is summarized from national catalogs of flora and species checklists in AlgaeBase [30]. The appearance of the microalga was mentioned in the United Kingdom, Ireland, Czechia, France, Germany, Ireland, The Netherlands, Norway, Portugal, Romania, Scandinavia, Slovakia, Spain and Sweden. In more detailed reports, the strains of *H. lacustris* were isolated from natural habitats. Allewaert et al. [110] obtained them from a water puddle on top of a container in Vlissingen (The Netherlands) as well as from a water puddle in a concrete depression and rainwater barrel in Ghent (Belgium). Dragoş et al. [112] isolated a *H. lacustris* strain from a sample of freshwater phytoplankton, collected from a fishpond near Cefa, Bihor District (Romania). Chekanov et al. [87,113] obtained a series of strains from the coastal zone of the Kandalaksha bay of the White Sea (Karelia, European part of Russia): from temporal rock ponds with semi-saline water, dry crust on the rocks and styrofoam sheets, biofilms in reddish water and water from the upper layer of a meromictic lake. Gacheva et al. [114] isolated the algae from an old granite bed of a dried fountain near Rozhen village (Blagoevgrad region, Bulgaria). Chelebieva et al. [115] reported the strain in the vicinity of the city of Sevastopol (Crimea, Ukraine), as well as from the European part of Russia (in the vicinity of Adler and mountainous region of the Central Caucasus). Some of *Haematococcus lacustris* are deposited to international culture collections, such as Scandinavian Culture Collection of Algae and Protozoa (SCCAP), Culture Collection of Algae at the University of Göttingen (SAG), Culture Collection of Algae at UT-Austin (UTEX), Algae Culture Collection of Kyiv University (ACKU), and Culture Collection of Autotrophic Organisms (CCALA). In particular, there are strains in SAG from Harz Mountains, bog pool at Bruchberg, 3000 m a.s.l. (Germany) isolated by Koch in 15959, from Aneboda (Sweeden) isolated by Pringsheim before 1966, from former Czechoslovakia, from pond Binai (Zbyny) near Hirschberg (Doksy), Bohemia, 1000 m a.s.l. (Czechia) isolated by Mainx before 1979, Botanical Garden of the University of Basel, Basel (Switzerland) isolated by Vischer in 1923, from roof of Botany School, Cambridge (United Kingdom) isolated by George in 1959 [116,117,118,119]. Several strains deposited to SAG were isolated by Zehnder in 1953: from the metallic holy water font and from the stony holy water font in the Eggenwil churchyard, isolated from Aargau (Switzerland), from the holy water font at the Sihlfeld church yard, Zürich (Switzerland), from little “blood pond” near Samnun, Graubünden (Switzerland) [116,119]. In CCALA, there are strains obtained from concrete, Březová nad Svitavou (Czechia) isolated by Ettl in 1958, from pond in Třeboň (Czechia) isolated by Takáčová in 1983, from pool in Veverská Bítýška (Czechia), isolated by Přibyl in 2005, from puddle in Brno (Czechia) isolated by Přibyl in 2009. A cold-tolerant strain of *Haematococcus lacustris* was isolated from Blomstrandhalvøya, Svalbard (Norway) [120].

An authentic strain of *H. rubens* was obtained from a water puddle on white bucket (Ghent, Belgium) [110]. The second known strain was isolated from supralittoral rock pool, Tvärminne (Finland) by Droop in 1951 [119]. Most strains of *H rubicundus* were obtained in Central Europe: from a water puddle on a white chair (Třeboň, Czechia), from a grey rain water barrel (Wageningen, The Netherlands), from water puddle on a white bucket and a rain water barrel (Ghent, Belgium), from a water puddle on green trash bin (Merelbeke, Belgium), from water puddle in white porcelain sink (Aaigem, Belgium) [110]. One was sampled from the dry crust on the rocks in the coastal zone of the Kandalaksha bay of the White Sea (Karelia, European part of Russia) [87]. One was isolated from the small water puddle in rock depression (Province of Pescara, Italy) [110]. The data on distribution of *Haematococcus* spp. in Europe are summarized in Table 2.

### 4.2. Ettlia Komárek (Chlorophyceae, Chlamydomonadales)

*Ettlia carotinosa* Komárek (Figure 4b) is one of the “non-canonical” carotenogenic green algae. Its former name is *Neochloris wimmeri* (Hilse) Archibald and Bold. Although it is considered as a member of a separate genus, in recent phylogenetic studies, it was shown that *Ettlia carotinosa* was close to the *Haematococcus* clade [121]. It was concluded that there is no need to transfer *Ettlia carotinosa* to the *Haematococcus* genus. Data on more strains of *Ettlia* are required. The predominant secondary carotenoid of the microalga is astaxanthin; however, an admixture of c.a. 10–15% adonirubin was observed [122] (Table 1). Xanthophylls are deposited mainly in the form of esters with fatty acids; astaxanthin monoesters are predominant [122]. There are also small amounts of canthaxanthin and β-carotene [122,123]. The maximal carotenoid content after carotenogenesis induction in *Ettlia carotinosa* is as high as 2.1% of the culture dry mass [124].

**Table 1:** Main secondary carotenoids accumulated in carotenogenic algae found in Europe.

Species	Phylum	Order	Predominant Secondary Carotenoid
*Acetabularia acetabulum*	Chlorophyta	Dasycladales	Astaxanthin
*Botryococcus braunii* race A	Chlorophyta	Trebouxiales	Astaxanthin
*Botryococcus braunii* race B	Chlorophyta	Trebouxiales	Echinenone, botryoxanthines, braunixanthines
*Botryococcus braunii* race L	Chlorophyta	Trebouxiales	Echinenone, and β-carotene
*Bracteacoccus aggregatus*	Chlorophyta	Sphaeropleales	Astaxanthin, adonirubin, and β-carotene
*Bracteacoccus bullatus*	Chlorophyta	Sphaeropleales	Echinenone and astaxanthin
*Bracteacoccus giganteus*	Chlorophyta	Sphaeropleales	Astaxanthaxanthin and canthaxanthin
*Bracteacoccus minor*	Chlorophyta	Sphaeropleales	Astaxanthin and canthaxanthin
*Chloromonas arctica*	Chlorophyta	Chlamydomonadales	NO DATA
*Chloromonas brevispina*	Chlorophyta	Chlamydomonadales	NO DATA
*Chloromonas rosae*	Chlorophyta	Chlamydomonadales	NO DATA
*Chloromonas rostafinskii*	Chlorophyta	Chlamydomonadales	NO DATA
*Chlainomonas rubra*	Chlorophyta	Chlamydomonadales	Astaxanthin
*Chloromonas nivalis*	Chlorophyta	Chlamydomonadales	Astaxanthin
*Chloromonas hindakii*	Chlorophyta	Chlamydomonadales	Astaxanthin
*Chloromonas krienitzii*	Chlorophyta	Chlamydomonadales	Astaxanthin
*Chlorosarcinopsis bastropiensis*	Chlorophyta	Chlamydomonadales	Canthaxanthin
*Chlorosarcinopsis dissociate*	Chlorophyta	Chlamydomonadales	Canthaxanthin
*Chromochloris zofingiensis*	Chlorophyta	Sphaeropleales	Astaxanthin and canthaxanthin
*Coelastrella rubescens*	Chlorophyta	Sphaeropleales	Mixture of xanthophylls ^1^ and α/β-carotene
*Coelastrella aeroterrestrica*	Chlorophyta	Sphaeropleales	Mixture of xanthophylls ^1^
*Coelastrella terrestris*	Chlorophyta	Sphaeropleales	Mixture of xanthophylls ^1^
*Coelastrella oocystiformis*	Chlorophyta	Sphaeropleales	Astaxanthin, canthaxanthin, β-carotene
*Deasonia granata*	Chlorophyta	Chlamydomonadales	Mixture of xanthophylls ^1,2^
*Diacronema vlkianum*	Haptophyta	Pavlovales	Astaxanthin
*Dunaliella salina*	Chlorophyta	Chlamydomonadales	β-carotene
*Ettlia carotinosa*	Chlorophyta	Chlamydomonadales	Astaxanthin + admixture of adonirubin
*Euglena rubida*	Euglenophyta	Euglenales	Astaxanthin
*Euglena sanguinea*	Euglenophyta	Euglenales	Astaxanthin and adonixanthin
*Golenkinia brevispicula*	Chlorophyta	Sphaeropleales	β-carotene + admixture of astaxanthin
*Haematococcus lacustris*	Chlorophyta	Chlamydomonadales	Astaxanthin
*Haematococcus rubicundus*	Chlorophyta	Chlamydomonadales	Astaxanthin
*Haematococcus rubens*	Chlorophyta	Chlamydomonadales	Astaxanthin
*Halochlorella rubescens*	Chlorophyta	Sphaeropleales	Canthaxanthin, astaxanthin, (β-carotene)
*Protosiphon botryoides*	Chlorophyta	Chlamydomonadales	Astaxanthin
*Pseudospongiococcum protococcoides*	Chlorophyta	Chlamydomonadales	Mixture of xanthophylls ^1^
*Sanguina aurantia*	Chlorophyta	Chlamydomonadales	Astaxanthin
*Sanguina nivaloides*	Chlorophyta	Chlamydomonadales	Astaxanthin
*Tovellia sanguinea*	Dinophyta	Gonyaulacales	Astaxanthin
*Tovellia rubescens*	Dinophyta	Gonyaulacales	Astaxanthin
*Trachelomonas volvocina*	Euglenophyta	Euglenales	Astaxanthin
*Tetraëdron minimum*	Chlorophyta	Sphaeropleales	Astaxanthin and adonixanthin
*Trentepohlia*	Chlorophyta	Trentepohliales	β-carotene
*Rhexinema sarcinoideum*	Chlorophyta	Ulvophyceae	Astaxanthin+ admixture of canthaxanthin

^1^ Mixture of three or more secondary xanthophylls in the amount of 10% of total pigment content; ^2^ based on unpublished data.

An authentic (and single known) strain *Ettlia carotinosa* SAG 213–4 (subcultures: CCAP 213/4, UTEX 113, ACKU 573–06) was isolated by Mainx from the soil sample collected in the vicinity of Praha (former Czechoslovakia) before 1954 (exact date is unknown). *Ettlia carotinosa* also was mentioned as *Neochloris wimmeri* in Germany [116,117,122,124] (Table 2). No other information about distribution of the species is available.

**Table 2:** Distribution of carotenogenic algae in Europe based on published data.

Species	Country and Region (If Available)
*Acetabularia acetabulum*	Mediterranean and Adriatic Seas
*Botryococcus braunii*	Cambridge (United Kingdom), Maddingley Brick Pits (United Kingdom) Cheshire (United Kingdom), Cumbria (United Kingdom), Brittany (France), Côte-d’Or, Morvan region (France), vicinity of Lingoult (France), Chaumecon Lake and Crescent Lake, Arcachon region (France), Large Lake of Sanguinet (France), barrier lake of Pareloup (France), Grasmere Lake, vicinity of Amieiro (Portugal); Ukraine, Ireland, The Netherlands, Norway, Poland, Romania, Russia ^1^, Spain, Sweden.
*Bracteacoccus aggregatus*	Yershovskoye Lake, Karelia (Russia ^1^); Czechia, Germany, Ukraine
*Bracteacoccus bullatus*	Sierra Nevada (Spain), Staro-Berdyansky forestland, Zaporozhye region (Ukraine), Dnipropetrovsk Oblast (Ukraine); Russia ^1^
*Bracteacoccus giganteus*	High Ardennes (Belgium); Germany, Russia ^1^, Ukraine
*Bracteacoccus minor*	Boreč Hill ventaroles (Czechia), Mykhailivska Tsilyna Nature Preserve, Katerynivka (Ukraine); Poland, Romania, Russia ^1^
*Chloromonas arctica*	Svalbard Archipelago (Norway) ^2^
*Chloromonas brevispina*	Tyrol Alps (Austria), Giant Mountains (Czechia), Svalbard Archipelago (Norway) ^2^
*Chlainomonas rubra*	Ľadové Lake in the High Tatras and Gossenkölle Lake in the Tyrolean Alps (Austria), Pirin Mountains (Bulgaria)
*Chloromonas rosae*	Tyrol Alps (Austria), Boreč Hill ventaroles (Czechia), Giant Mountains (Czechia)
*Chloromonas rostafinskii*	Stara Planina, Central Balkan Mountains (Bulgaria)
*Chloromonas nivalis*	Alps, Tyrol (Austria), Zheleznitza village, Vitosha Mountains (Bulgaria), Pirin Mountains (Bulgaria), Stara Planina, Central Balkan Mountains (Bulgaria), High Tatra Mountains (Slovakia), Sierra Nevada Mountains (Spain), Pyrenees (Spain, France), Giant Mountains (Czechia), Jeseníky Mountains (Czechia), Mountain Olympus (Greece), Svalbard Archipelago (Norway) ^2^
*Chloromonas hindakii*	High Tatra Mountains (Slovakia, Poland), Krkonoše and Jeseníky Mountains (Czechia)
*Chloromonas krienitzii*	Sarntal Alps, South Tyrol (Italy), High Tatra Mountains (Slovakia, Poland), North Pindus (Greece)
*Chlorosarcinopsis bastropiensis*	Ukraine
*Chlorosarcinopsis dissociata*	Snake Islands Tract, Kanevsky Natural Reserve, Cherkasy Oblast (Ukraine)
*Chromochloris zofingiensis*	Ram Oswald near Zofingen (Switzerland), Dalmatia (Former Yugoslavia), Unterengadin (Switzerland), Ortenberg near Marburg/Lahn (Germany), Firenze (Italy), Sklene Teplice (Slovakia); Iceland, Croatia, France, Romania, Bulgaria, Russia ^1^
*Coelastrella rubescens*	Pitschberg mountain, South Tirol (Austria), South Tirol (Italy), Rastorguevo Village, Moskovskaya Oblast (Russia ^1^)
*Coelastrella aeroterrestrica*	Pirin Mountains (Bulgaria), Obergurgl, Tirol (Austria), Kandalaksha bay of the White Sea, Karelia (Russia ^1^), Odesa Oblast (Ukraine); The Netherlands.
*Coelastrella terrestris*	Sölheimjökull glacier (Iceland), Obergurgl, Tyrol (Austria), Pirin Mountains (Bulgaria), Odesa Oblast (Ukraine); Czechia, Germany, Italy, The Netherlands, Poland, Russia ^1^, Slovakia, Romania
*Coelastrella oocystiformis*	Windermere (United Kingdom), Boreč Hill ventaroles (Czechia)
*Deasonia granata*	Praha (Czechia), Gomel (Belarus)
*Diacronema vlkianum*	English Channel (France), sea water Ryde, Isle of Wight, England (United Kingdom). Sea coast (Portugal), Kühnhausen near Erfurt (Germany); Ireland, Portugal, Romania, Spain
*Dunaliella salina*	Salt Lake Elton, Volgograd Oblast (Russia ^1^), Razval, Orenburg Oblast (Russia ^1^), Arinaga Saltwork, Monzon (Spain), Gran Canaria (Spain). Kuyalnitsky Liman, Odesa oblast (Ukraine), Zmievo Lake, sedimentation pond of Heroyskoe salt works, Kherson Oblast (Ukraine), Genicheskoe Lake, sedimentation pond of Genichesk salt works, Kherson Oblast (Ukraine), IBSS Siwash bay, Crimea (Ukraine), Filatovskaya salt flat, Crimea (Ukraine), Sasyk Lake, Crimea (Ukraine), Saki, Crimea (Ukraine), Lacul Sărat (Romania); Germany, Portugal
*Ettlia carotinosa*	Vicinity of Praha (Czechia); Germany
*Euglena rubida*	Branicki Palace, Białystok (Poland)
*Euglena sanguinea*	Eichenbirkig, Fränkische Schweiz, Bayern (Germany), Spydeberg, Ehrenberg, the county of Østfold (Norway), vicinity of Debden (United Kingdom); Denmark, Belarus, Bulgaria, Czechia, Estonia, Hungary, Italy, Latvia, Moldova, The Netherlands, Poland, Romania, Slovakia, Spain, Ukraine
*Golenkinia brevispicula*	Vicinity of Dortmund (Germany), The Netherlands
*Haematococcus lacustris*	Rozhen village (Bulgaria), Ghent (Belgium), Cefa, Bihor District (Romania), Bruchberg (Germany), Aneboda (Sweden), Basel (Switzerland), Aargau (Switzerland), Zürich (Switzerland), Graubünden (Switzerland), Zbyny, Hirschberg (Czechia), Březová nad Svitavou (Czechia), Třeboň (Czechia), Veverská Bítýška (Czechia), Brno (Czechia), Sevastopol, Crimea (Ukraine), Vlissingen (The Netherlands), Svalbard Archipelago (Norway) ^2^, coastal zone of the Kandalaksha bay of the White Sea, Karelia (Russia ^1^), vicinity of Adler and mountainous region of the Central Caucasus (Russia ^1^), Cambridge (United Kingdom); Ireland, France, Norway, Portugal, Slovakia, Spain
*Haematococcus rubicundus*	Třeboň (Czechia), Wageningen (The Netherlands), Ghent (Belgium), Merelbeke (Belgium), Aaigem (Belgium) Kandalaksha bay of the White Sea, Karelia (Russia ^1^), Province of Pescara (Italy)
*Haematococcus rubens*	Ghent (Belgium), Tvärminne (Finland)
*Halochlorella rubescens*	Vicinity of Bordeaux (France), Buhta Blagopoluchiya, Bolshoy Slovetskii Island (Russia ^1^), Lake Solone, Zaporizhzhya Oblast (Ukraine)
*Protosiphon botryoides*	Samara forest, Dnepropetrovsk Region (Ukraine), Františkovy Lázně (Czechia), Lützel-Breitenborn (Germany); United Kingdom, Ireland, Portugal, Spain
*Pseudospongiococcum protococcoides*	Windermere (United Kingdom), Arabat Spit, Crimea (Ukraine)
*Sanguina aurantia*	Svalbard Archipelago (Norway) ^2^
*Sanguina nivaloides*	Ötztal Alps (Austria), Sarntal Alps (Italy), High Tatra Mountains (Slovakia), Alps (Slovenia), Urner Alps (Switzerland); Svalbard Archipelago (Norway) ^2^; Norway
*Tetraëdron minimum*	Þórsmörk (Iceland); United Kingdom, Bulgaria, Czechia, France, Germany, Ireland, The Netherlands, Norway, Portugal, Romania, Russia ^1^, Slovakia, Spain, Sweden, Ukraine
*Tovellia sanguinea*	Trentino Province (Italy)
*Tovellia rubescens*	Gafanha da Boavista, Ílhavo (Portugal)
*Trachelomonas volvocina*	United Kingdom, Poland; Ireland, Bulgaria, Czechia, Germany, The Netherlands, Romania, Russia, Scandinavia, Slovakia, Spain, Sweden, Ukraine
*Trentepohlia* spp.	Ubiquitous in Europe
*Rhexinema sarcinoideum*	Institute of Soil Science and Plant Cultivatio near Puławy (Poland), Chelčice, South Bohemia (Czechia); Ukraine, Russia

^1^ Only European part of Russia was considered; ^2^ Formally not related to continental Europe.

### 4.3. Dunaliella Teodoresco (Chlorophyceae, Chlamydomonadales)

*Dunaliella salina* (Figure 4c) was also previously known as *Dunaliella bardawil* Ben-Amotz and Avron. Although current systematics of the genus *Dunaliella* is far from a well-established state [10], the name *Dunaliella salina* is commonly accepted to denote halophilic carotenoid producing *Dunaliella*. This species accumulates exclusively β-carotene as a secondary carotenoid [1,9,54] (Table 1).

According to AlgaeBase [30], *Dunaliella salina* was mentioned in Ukraine, Germany, Portugal, Romania, European parts of Russia, and Spain (Table 2). Recently the strains were isolated from salt ponds of Monzon (Spain) [125], from salt lakes Elton, Volgograd Oblast (European part of Russia), and Razval, Orenburg Oblast (European part of Russia) [126,127]. The strain CCAP 19/39 was obtained from a sea salt sample from Arinaga Saltwork, Gran Canaria (Spain) (Table 2). The alga was isolated from Lacul Sărat (Romania) [128]. It is plhylogenetically close to *Dunaliella salina* [127] isolated and deposited to the SAG collection [116]. Many strains were isolated in Ukraine: from Zmievo Lake (Kherson Oblast), from a sedimentation pond of Heroyskoe salt works (Kherson Oblast), from Genicheskoe Lake (Kherson Oblast), from sedimentation pond of Genichesk salt works (Kherson Oblast), from IBSS Siwash Bay (Crimea), from Filatovskaya salt flat bay, behind the dam of salt works of soda plant (Crimea), and from Sasyk Lake, sedimentation pond of salt works of cooperative “Halite” (Crimea), from evaporation ponds, Saki (Crimea) [37,129], from Kuyalnitsky Liman (Odesa Oblast) [130]. The presence of the microalga also was noticed on the Arabat Spit, Crimea (Ukraine) [131] (Table 2).

### 4.4. Chromochloris Kol and Chodat (Chlorophyceae, Chlamydomonadales)

*Chromochloris zofingiensis* (Dönz) Fucíková and Lewis (Figure 4d) is also known under the synonyms *Chlorella zofingiensis* Dönz, *Muriella zofingiensis* (Dönz) Hindák, *Mychonastes zofingiensis* (Dönz) Kalina and Puncochárová, *Chromochloris cinnabarina* Kol and Chodat, *Bracteacoccus cinnabarinus* (Kol and Chodat) Starr, and *Bracteacoccus minutus* Schwarz [29,30,132,133]. It is widely studied as a possible industrial source of carotenoids [16,83,134,135,136,137]. Its main secondary carotenoids are astaxanthin (predominantly in the form of monoesters with fatty acids) and echinenone [62,123,133,136,138] (Table 1). Astaxanthin content varies from 0.1 to 1.3% of cell dry mass depending on culturing conditions [134,139]. Significant amount of adonixanthin (predominantly in the form of diesters with fatty acids) is also observed [136]. Notably, *Chromochloris* is characterized by a high content of free astaxanthin (compared to other carotenogenic algae) [136]. It could be explained by independence of astaxanthin and fatty acid biosynthesis in this microalga [140]. This distinguishes *Chromochloris zofingiensis* from *Haematococcus lacustris*, where cerulenin, an inhibitor of fatty acid biosynthesis, blocks astaxanthin accumulation [141].

There are some data on isolated European strains of *Chromochloris zofingiensis*. For example, they have been deposited to SCCAP, SAG, UTEX, ACKU, and CCALA. They were isolated from soil samples from Ram Oswald near Zofingen, 1000 m a.s.l. (Switzerland) [132], from soil of Dalmatia (Former Yugoslavia) [132], from soil of Unterengadin, 8000 m a.s.l. (Switzerland) [142], from sample from cave wall (France) (unpublished data, GenBank accession numbers OK217227.1, FN597652.1), from a bark of a deciduous tree, Ortenberg near Marburg/Lahn (Germany) by Czygan in 1963 [116] (Table 2). The strains isolated from Firenze (Italy) and Sklene Teplice (Slovakia) were deposited to CCALA (GenBank accession number is MW075310.1). *Chromochloris zofingiensis* was mentioned in Slovakia, Romania, and the European part of Russia [30] (Table 2).

### 4.5. Chloromonas Gobi (Chlorophyceae, Chlamydomonadales)

The snow microalgae *Chloromonas nivalis* (Chodat) Hoham and Mullet, *Chloromonas krienitzii* Matsuzaki and Nozaki (Figure 4e) and *Chloromonas hindakii* Procházková and Remias accumulate secondary carotenoids [51,76,84,107,143,144,145]. The genus *Chloromonas* should also include “*Scotiella cryophila* Chodat” found in Austrian Alps (Tyrol, district Imst at Kühtai Valley, between Schwarzmoos and Gossenkö lle Lake) [146]. Predominant carotenoid of studied *Chloromonas* spp. is astaxanthin [51,76,84,107,143,144,145] (Table 1). In *Chloromonas nivalis* it is deposited mainly in the form of fatty acid monoesters [147] and diglycosides with fatty acid radicals [51]. Astaxanthin content in this microalga is c.a. 20.85 mg per 1 mg of chlorophyll *a* [107].

Distribution of *Chloromonas* spp. microalgae has been thoroughly reviewed by Hoham and Remias [73]. *Chloromonas nivalis* inhabits alpine snow. It causes appearance of red or in some cases green-, brownish-, orange- or pink-colored snow [73,143,145,148,149,150]. This species also includes previously described *Scotiella tatrae* Kol (currently *Chloromonas nivalis* subsp. *tatrae* Procházková, Remias, Řezanka and Nedbalová) [144,151] It was isolated from snow samples at Kühtai, in the proximity of Gossenkölle Lake, Tyrol Alps (Austria) [143], from snow samples in the vicinity of Zheleznitza village, at the edge of timber line, c.a. 1900 m a.s.l., Vitosha Mountains (Bulgaria) [152], from a shore of Capie Lake [151], from a shore and ice cover of Okrúhle Lake [153] in High Tatra Mountains (Slovakia) [144], from Tyrol, Alps (Austria) [107], from Sierra Nevada Mountains (Spain) [154], from Giant Mountains (Czechia) at altitudes 730–1545 m a.s.l. [149], on Tiefenbach Glacier, Alps, Tyrol, 2980 m a.s.l. (Austria) [155], in Kühtai, 2300 m a.s.l., Mountain Schönwieskopf, near Obergurgl, Ötztal, 2350 m a.s.l. (Austria) [156], in snow fields persisted in the Stara Planina, Central Balkan Mountains (Bulgaria) [157], Pirin Mountains, 1996–2930 m a.s.l. (Bulgaria) [51,158], on the Mountain Olympus, different altitudes (Greece) [159], the Pyrenees (France, Spain) [76,77], Jeseníky Mountains (Czechia) [144] (Table 2). Another cryophylic caotenogenic microalga, *Chloromonas hindakii*, was found in the samples of snow from the High Tatra Mountains (Slovakia, Poland), Krkonoše, and Jeseníky Mountains (Czechia) [144] (Table 2). *Chloromonas krienitzii* was sampled from the snow in the Sarntal Alps, South Tyrol (Italy), High Tatra Mountains (Slovakia, Poland) and from North Pindus (Greece) [145] (Table 2). Procházková et al. [160] described *Chloromonas kaweckae* Procházková, Matsuzaki, Řezanka, Nedbalová and Remias in the High Tatras (Slovakia) tolerant to high light intensities. Diversity of *Chloromonas* was also studied in the Svalbard Archipelago (Norway) [73] (Table 2).

There are also reports about other snow carotenogenic *Chloromonas* spp. (*Chloromonas brevispina* (Fritsch) Hoham, Roemer and Mullet, *Chloromonas rosae* (H. and O. Ettl) Ettl, *Chloromonas rostafinskii* (Starmach and Kawecka) Gerloff and Ettl), and *Chloromonas arctica* Barcyte and Hodač [73,149,156,157,158,161,162] (Table 2), their carotenoid composition is poorly studied. It should be noted that, although the samples of snow algae were collected and reported, in most cases there is no information that they were deposited to culture collections as strains. Only a few carotenogenic strains have been deposited. For example, *Chloromonas rosae* from High Tatra Mountains is stored in SAG, ACKU and UTEX [116,117].

### 4.6. Chlainomonas Christen (Chlorophyceae, Chlamydomonadales)

*Chlainomonas rubra* (Stein and Brooke) Hoham is another species of European snow carotenogenic algae. Its predominant carotenoid is astaxanthin. Procházková et al. [85] studied pigment composition on *Chlainomonas* from High Tatras and Austrian Alps populations (Figure 4f). Their secondary carotenoid contents were 93.0% and 88.5% of the total pigment pool, respectively, with astaxanthin as a predominant carotenoid (Table 1). Astaxanthin content was 44.5% and 31.9% of the total pigment pool in the High Tatra Mountains and Austrian Alps, respectively. Most likely, astaxanthin was deposited mainly in the esterified form [85]. Remias et al. [147] studied Austrian strains *Chlainomonas* sp. DR53 and *Chlainomonas* sp. AS02. Their predominant carotenoid was also astaxanthin. It was deposited predominantly in the form of diesters with fatty acids (but not their glycosides) [147].

Sumarizing the data on *Chlainomonas* distribution in Europe, *Chlainomonas* sp. was sampled in the vicinity of Gossenkölle lake, Tyrol, 2416 m a.s.l. (Austria), Hallstätter Glacier and Upper Austria (Austria) [147]. *Chlainomonas rubra* samples were collected in the Ľadové Lake in the High Tatras and Gossenkölle Lake in the Tyrolean Alps (Austria) [85], Tyrol, Alps [163] and Pirin Mountains, 1996–2930 m a.s.l. (Bulgaria) [158] (Table 2). It seems to be, there are currently no carotenogenic strains deposited into public collections of international value. 

### 4.7. Sanguina Leya, Procházková and Nedbalová (Chlorophyceae, Chlamydomonadales)

Snow algae *Sanguina nivaloides* Procházková, Leya and Nedbalová (Figure 4g) and *Sanguina aurantia* Leya, Procházková and Nedbalová were recently described [86]. They are responsible for the red and orange coloration of snow, respectively [86]. The genus *Sanguina* is close related to *Chloromonas* [86]. Astaxanthin is the most abundant secondary carotenoid of *Sanguina* (72 ± 9.9% and 91.7 ± 0.9% of all pigments in *Sanguina aurantia* and *Sanguina nivaloides*, respectively) [164] (Table 1). *Sanguina nivaloides* is characterized by cosmopolitan distribution, whereas *Sanguina aurantia* has been found in Arctic and Subarctic regions [86].

The algae were collected from samples of snow from Haferkarlespitze, High Tauern, Kühtai, Ötztal Alps (Austria), from Dolomites, Sarntal Alps (Italy), from High Tatra Mountains (Slovakia), from Alps (Slovenia) and from Urner Alps (Switzerland) [86] (Table 2). Only *Sanguina nivaloides* was found in continental Europe by Procházková et al. [86], *Sanguina aurantia* was sampled only on the Svalbard Archipelago (Norway) (Table 2). No strains of *Sanguina* are available in culture, and hence, their life cycles, which should include migrating flagellates, are unknown [164].

### 4.8. Coelastrella Chodat (Chlorophyceae, Sphaeropleales)

Members of the genus *Coelastrella* are also known as carotenoid producers. Carotenogenic alga *Coelastrella rubescens* (Vinatzer) Kaufnerová are Eliás [70,165] (Figure 4h) was previously related to the genus *Scotiellopsis*, therefore also known under a former synonym *Scotiellopsis rubescens* Vinatzer [166]. The same is true about *Coelastrella terrestris* (Reisigl) Hegewald and N.Hanagata [166], which also accumulates carotenoids [167]. Carotenogenesis also has been reported for European strains of *Coelastrella aeroterrestrica* Tschaikner, Gärtner and Kofler [87]. *Coelastrella* spp. are characterized by a diverse composition of secondary carotenoids. *Coelastrella rubescens* contains comparable amounts of different ketocarotenoids (adonixanthin, echinenone, canthaxanthin, astaxanthin) as well as α-carotene and β-carotene [70,165] (Table 1). *Coelastrella aeroterrestrica* [87] and *Coelastrella terrestris* [167] accumulate a mixture of secondary xanthophylls. *Coelastrella oocystiformis* (Lund) Hegewald and Hanagata (formerly *Scotiellopsis oocistiformes* Lund) accumulates predominantly astaxanthin with admixtures of β-carotene and canthaxanthin [123,168] (Table 1). Astaxanthin content under nitrogen starvation and high light in this species is c.a. 1% of cell dry mass [168].

*Coelastrella* spp. are aeroterrestrial and soil algae, they are found in peat pools, *Sphagnum* beds, as a crust on biotic or abiotic surfaces, where they form orange or reddish colonies. Characterized strains of *Coelastrella rubescens* were isolated from soil on a Pitschberg mountain, 2300 m a.s.l., South Tirol (Austria) [169], from soil in South Tirol (Italy) [170] and from the surface of bark from an apple tree (*Malus × domestica*) in Rastorguevo Village, Moskovskaya Oblast (European part of Russia) [70] (Table 2). 

*Coelastrella terrestris* was noted in Europe: Germany, Italy, The Netherlands, Poland, European part of Russia, Slovakia, and Romania [30]. The strains of *Coelastrella terrestris* were isolated in the foreland of Sölheimjökull glacier from a small brook “where macroscopic mucilaginous mats of reddish cyanobacteria dominated” (Iceland) [167], from soil in Czechia [171], from alpine soil near the village Obergurgl, Tyrol (Austria) [172], and from soils in Pirin Mountains (Bulgaria). It was found in the soil crusts from sand dunes of the Danube Delta, Odesa Oblast (Ukraine) [173] (Table 2).

*Coelastrella aeroterrestrica* was reported from soils in Pirin Mountains (Bulgaria) [174], soils of Alpine grassland and ski slopes near the village Obergurgl, Tirol (Austria) [175], from reddish crust on a piece of styrofoam, Kandalaksha bay of the White Sea, Karelia (European part of Russia) [87]. This species also was mentioned in The Netherlands [30]. It was found in the soil crusts from sand dunes of the Danube Delta, Odesa Oblast (Ukraine) [173] (Table 2). 

The strain *Coelastrella* sp. S6 [176] isolated from an open pond in the Liège region (Belgium), *Coelastrella* sp. BGV from a metal tub found in the village Varvara (Bulgaria) [177] and the strain *Coelastrella* sp. FGS-001 sister to the clade *C. thermophila* var. *globulina* Song, Liu, Liu and Hu from a foliose, land-living colony of *Nostoc commune* in Ås, Akershus County (Norway) [178] (Table 2). 

An authentic strain of *Coelastrella oocystiformis* was isolated from a rock face near the Freshwater Biological Associaton’s laboratory in Windermere (United Kingdom) by Fogg before 1957 (exact date is unknown) [116]. Its distribution was studied in Boreč Hill ventaroles (Czechia) [162] (Table 2).

### 4.9. Bracteacoccus Tereg (Chlorophyceae, Sphaeropleales)

Three strains of *Bracteacoccus* have been reported producing carotenoids: *Bracteacoccus minor* (Schmidle ex Chodat) Petrová [133,179,180], *Bracteacoccus giganteus* Bischoff and Bold [180], *Bracteacoccus aggregatus* Tereg (former synonym *Bracteacoccus cohaerens* Bischoff and Bold) (Figure 4i) with the maximal carotenoid content of 3.0% of cell dry mass [181], and *Bracteacoccus bullatus* Fuciková Flechtner and Lewis [182]. In *Bracteacoccus minor* and *Bracteacoccus giganteus*, astaxanthin is a predominant carotenoid with an admixture of canthaxanthin [133,179,180] (Table 1). Its total carotenoid content reaches 1.0% of cell dry mass [179]. *Bracteacuccus aggregatus* accumulates a mixture of astaxanthin, adonirubin and β-carotene [181]. *Bracteacoccus bullatus* contains echinenone and astaxanthin [182] (Table 1). As a rule, *Bracteacoccus* spp. accumulate hydroxylated xanthophylls mainly in the form of esters with fatty acids with a predominance of astaxanthin diesters [179,181], but *Bracteacoccus giganteus* accumulate monoesters predominantly [180].

Carotenogenic strains of *Bracteacoccus aggregatus*, *Bracteacoccus bullatus*, *Bractracoccus giganteus*, and *Bracteacoccus minor* were found in Europe. *Bracteacoccus bullatus* and *Bracteacoccus aggregatus* were noted in Czechia, Germany, European part of Russia, and Ukraine [30]. Carotenogenic strain of *Bracteacoccus aggregatus* was isolated from the water of the Yershovskoye Lake (the flow through) with the salinity of 6‰ in the coastal zone of the Kandalaksha bay of the White Sea, Karelia (European part of Russia) [87] (Table 2). The strain of *Bracteacoccus minor* was obtained from a snow sample in Sierra Nevada Mountains (Spain) [182]. The microalga was also found in the Wyżyna Krakowsko Wieluńska upland, it was found in Cave Łabajowa, Cave Żarska, Cave Głęboka, Cave Zbójecka, Cave Ciemna, and Cave Pustelnia (Poland) [183] and in caves Grott de Remouchamps and Grotte gauche de Fonds de Forêt (Belgia) [184]. Kostikov et al. noted these algae in Carpathians and Crimean Mountains (Ukraine) [185]. It was found in Mykhailivska Tsilyna Nature Preserve, Katerynivka (Ukraine) [186] (Table 2). Distribution of *Bracteacoccus minor* was studied in Boreč Hill ventaroles (Czechia) [162]. The strain of *Bracteacoccus giganteus* was isolated from the acidified brown soil in spruce forest, experimental site Waroneu, High Ardennes (Belgium) by Kostikov in 1996 and deposited to ACKU [117] (Table 2). Carotenogenic strain of Bracteacoccus bullatus was isolated from a snow sample, Sierra Nevada (Spain) by Cepák in 2010 and deposited to CCALA [154,182], from the locust plantation of the Staro-Berdyansky forestland, Zaporozhye region (Ukraine) [187], and from the Robinia forest, Dnipropetrovsk region (Ukraine) [188] (Table 2).

### 4.10. Halochlorella Dangeard (Chlorophycee, Sphaerapleales)

*Halochlorella rubescens* Dangeard is also known under former synonyms *Chlorella emersonii* var. *rubescens* (Dangeard) Fott, Lochead and Clemençon, and *Chlorella fusca* var. *rubescens* (Dangeard) Kessler, Czygan, Fott and Nováková) (Figure 4j) It is well known as a carotenoid-accumulating organism. Carotenoid composition and content for this alga depends on strain and/or culturing conditions. Jo et al. [189] reported that the main secondary carotenoids were canthaxanthin and astaxanthin (Table 1). Their contents after carotenogenesis induction are c.a. 1.8 and 1.2% of cell dry mass, respectively. In another work, carotenoid content was 1.9–2.2% of cell dry mass with the predominance of β-carotene, astaxanthin, and canthaxanthin [190]. 

The data on distribution are scarce. The authentic strain SAG 5.95 (subcultures ACKU 647–06, CCAP 232/1) [116,117] was isolated from a culture of brown alga near Bordeaux (France) [191] (Table 2). One strain was isolated from the sample of water from the littoral of the Buhta Blagopoluchiya Bay, Bolshoy Slovetskii Island (European part of Russia), GenBank accession numbers OP810940.1 and OP810416.1 [88] (Table 2). It was also mentioned on the coast of the Salt Lake Solone, Zaporizhzhya Oblast (Ukraine) [192] (Table 2).

### 4.11. Tetraëdron Kützing (Chlorophyceae, Sphaeropleales)

The microalga *Tetraëdron minutum* (Braun) Hansgirg (Figure 4k) has been recently reported as a carotenoid producer. This unicellular chlorophyte is a part of phytoplankton, can be adhered to submerged surfaces. It inhabits ponds and small lakes [30]. After induction by a salt stress (c.a. 150 Pa), it accumulates 61.1% astaxanthin and 38.9% adonixanthin as secondary carotenoids [193] (Table 1).

The microalga *Tetraëdron minimum* is commonly distributed in small freshwater ponds and in seawater. It was mentioned in the United Kingdom, Ireland, Bulgaria, Czechia, France, Germany, Ireland, The Netherlands, Norway, Portugal, Romania, European part of Russia, Slovakia, Spain, Sweden, and Ukraine [30] (Table 2). A single strain with described carotenoid accumulation was isolated from a wet grave by dripping water close to a steep, sun-exposed slope at Þórsmörk (Iceland) [193] (Table 2).

### 4.12. Deasonia Ettl and Komárek (Chlorophyceae, Chlamydomonadales)

*Deasonia* is a genus of green microalgae poorly studied in terms of carotenogenesis. The fact of carotenoids accumulation has been noticed for *Deasonia granata* [194] and the strain *Deasonia* sp. NAMSU 934/2 [90] (Figure 4l). No data have been published on the carotenoid composition of these strains. Based on the absorbance spectra of pigment extracts [90], *Deasonia* sp. NAMSU 934/2 accumulates ketocarotenoids under stress conditions. Its predominant carotenoid seems to be astaxanthin (unpublished data).

The strain *Deasonia* sp. CALU 934 was isolated from the soil samples on the shore of a lake in the Gvardeyskoye Settlement, Leningrad Oblast, European part of Russia [90,195]. The strain defined as *Deasonia granata* ACKU 566–06 (authentic strain of the genus) was isolated by Pringsheim from soil in the vicinity of the city of Praha (former Czechoslovakia) [116,117], the strain *Deasiona garanata* ACSSI 150 was isolated from the soil in Gomel (Belarus) [196] (Table 2).

### 4.13. Chlorosarcinopsis Herndon (Chlorophyceae, Chlamydomonadales)

The microalgae from the *Chlorosarcinopsis* genus are considered as carotenoid-producing, although data on carotenoid composition is poor. They produce a mixture of ketocarotenoids with a predominance of canthaxanthin [91] (Table 1). Carotenogenesis was mentioned in *Chlorosarcinopsis bastropiensis* Groover and Bold [91,197] (Figure 4m) and *Chlorosarcinopsis dissociata* Herndon [197,198] inhabiting Europe.

*Chlarosarcinopsis dissociata* and *Chlorosarcinopsis bastropiensis* were mentioned in Ukraine, especially in Mountain Crimea [30,199]. Two strains of *Chlorosarcinopsis dissociata* were isolated from the forest soil in the Snake Islands Tract, Kanevsky Natural Reserve, Cherkasy Oblast (Ukraine) by Demchenko and deposited to ACKU [117] (Table 2). The strains of this genus were isolated from the soil samples in Ukraine. The strain of the microalga of unknown origin was deposited to ACKU [117]. Several strains defined as *Chlorosarcinopsis* sp. isolated from different sites in Ukraine also were deposited to the same collection [117]. No data on European strains of this species in other collections (Table 2).

### 4.14. Acetabularia Lamouroux (Ulvophyceae, Dasycladales)

Little is known about the carotenoid accumulating *Acetabularia acetabulum* (L.) Silva previously known as *Acetabularia mediterranea* Lamouroux. In fact, the siphonal macroscopic thallus of *Acetabularia* is represented by one large uninucleate cell with a complex shape (Figure 4n). Accumulation of astaxanthin by this organism has been reported in two works: in 1967 and in 1986 [200,201]. Since that time, this topic has been of no interest to the researchers. Besides astaxanthin, it also contains 3-hydroxyechinenone, adonixanthin, adonirubin and “unknown yellow pigment” [200] (Table 1). Currently, *Acetabularia* is not considered as a source of carotenoids. However, it could serve as a source of astaxanthin for marine animals eating algae in the wild [92,202].

*Acetabularia acetabulum* is a marine alga distributed in the Mediterranean and Adriatic Seas [30]. The presence of astaxanthin has been recorded for the samples obtained in the Adriatic Sea, Sipan Island, in the vicinity of Dubrovnik (former Yugoslavia) [201] and in the Stazione Zoologica Naple (Italy) (Table 2).

### 4.15. Pseudospongiococcum Gromov and Mamkaeva (Chlorophyceae, Chlamydomonadales)

*Pseudospongiococcum protococcoides* Gromov and Mamkaeva (Figure 4o) is a little-known species of unicellular algae with the ability to accumulate secondary carotenoids [93,203]. The strain was proposed as a potential source of these pigments. Its total carotenoid content reaches 0.4% of cell dry mass, which is relatively low. However, it is characterized by high growth rate, therefore is considered as a prospective carotenoid producer [93]. It accumulates predominantly ketocarotenoids [203] (Table 1).

Single known strain of *Pseudospongiococcum protococcoides* CALU 221 (GenBank accession numbers (MZ126559.1, KU057947.1) was isolated from rock face near the Freshwater Biological Associaton’s laboratory in Windermere (United Kingdom) by Fogg in 1957 [204] (Table 2). No data about distribution. In one report, the microalga was also noticed on the Arabat Spit, Crimea (Ukraine) [131] (Table 2).

### 4.16. Protosiphon Klebs (Chlorophyceae, Chlamydomonadales)

Secondary carotenogenesis were studied in *Protosiphon botryoides* (Kützing) Klebs [205] (Figure 4p). Its total carotenoid content is c.a. 1.7% of cell dry mass [168]. The main secondary carotenoid is astaxanthin in the form of mono- and diesters with fatty acids [123,168] (Table 1). As in most other carotenogenic microalgae, induction of carotenogenesis in previous studies was induced by nitrogen starvation and high light [168].

Carotenoid producing *Protosiphon botryoides* is a soil alga. Its strains were isolated from the soil of the floodplain birch-ash grove of the Samara forest, Dnepropetrovsk Region (Ukraine) [206], from soil in Františkovy Lázně, 5500 m (Czechia) by Pringsheim and from soil from massdevelopment on field on red sandstone, Hessen, Biebergemuend/Spessart, OT Lützel-Breitenborn (Germany) [116], from soil (Czechia) [171] (Table 2). It was also noticed in the United Kingdom, Ireland, Czechia, Portugal, Spain [30] (Table 2).

### 4.17. Botryococcus Kützing (Trebouxiophyceae, Trebouxiales)

*Botryococcus braunii* is a unicellular alga forming colonia with botryoid organization (Figure 4q). It is able to accumulate secondary carotenoids [95,207,208,209]. Its strains are divided into three different races, namely A, B, and L, distinguished in terms of accumulated secondary metabolites [209,210,211]. These races are characterized by different carotenoid profiles [209,210,212]. Only race A accumulates astaxanthin (Table 1), whereas the biomass of races B and L is enriched by echinenone. Race L is characterized by an increased amount of β-carotene, whereas race B contains a higher amount of echinenone (up to 73% of total carotenoid content) (Table 1). In addition, race B accumulates botryoxanthines A and B, α-botryoxanthine (10–11% of total carotenoids), as well as braunixanthines 1 and 2 (23% of total carotenoids) (Table 1). Biomass of race A is also enriched by the primary carotenoid lutein. It seems to be that secondary carotenoids are deposited in *Botryococcus* cells in lipid bodies as in other carotenogenic algae, but it can excrete them from the cytoplasm to the extracellular matrix by the external pressure [211] (Figure 4q). It can facilitate pigment extraction. Total carotenoid content in *Botryococcus braunii* at the end of the culturing varies from 0.25% to 0.55% of cell dry mass depending on strain and culturing conditions [208,210,212], that is lower than in some other carotenogenic representatives, such as *Haematococcus*, *Dunaliella*, *Coelastrella*, *Pseudospongiococcum*, and *Bracteacoccus* (see above).

The species is abundant in brackish lakes, reservoirs, and freshwater bodies [209]. The strains of *Botryococcus braunii* were isolated from Madingley Brick Pits, Cambridge (United Kingdom) [213,214], from an extensive orange bloom floating on the surface of Oakmere, Cheshire, England (United Kingdom) [215], from water samples of the lake of Coat ar Herno, in Brittany (France) [216], a barrier lake near Grosbois-en-Montagne, Côte-d’Or, Morvan region (France) [217]. Samples were also taken from a small pool near Lingoult, two barrier lakes, Chaumecon and Crescent in Arcachon region, Large Lake of Sanguinet, Barrier Lake of Pareloup (France) [218], from Grasmere Lake, Cumbria (United Kingdom), and from a small pool near Amieiro (Portugal) [219,220]; one strain was isolated in Ukraine [221] (Table 2). It was noted in Ireland, Czechia, Germany, Ireland, The Netherlands, Norway, Poland, Romania, the European part of Russia, Spain, Sweden [30] (Table 2). *Botryococcus braunii* is widespread in water bodies in Ukraine [222] (Table 2).

### 4.18. Golenkinia Chodat (Chlorophycee, Sphaerapleales)

Rearte et al. [96] studied the microalgae *Golenkinia* aff. *brevispicula* Hegewald and Schnepf (Figure 4r) as a possible source of carotenoids. Under inductive conditions accumulation of β-carotene with an admixture of astaxanthin (c.a. 10% of total carotenoids) is observed (Table 1). Maximal carotenoid content is c.a. 0.25% of culture dry mass. In a single found work on carotenoid production in the strain FAUBA-3 of *Golenkinia* aff. *brevispicula* [96], salt stress (osmotic pressure c.a. 3 kPa) was used for induction of carotenogenesis. It seems that the stress effect was not sufficiently intensive. Indeed, effective carotenoid accumulation is accompanied by a strong reduction in PSA and photosynthetic activity [223]. However, it was not the case for *Golenkinia* aff. *brevispicula* under salt stress: significant amounts of primary carotenoids (lutein, violaxanthin, and zeaxanthin) and non-zero parameters of primary photochemistry [96]. Most likely, low yield of the pigments can be improved by enhancing the stress, for example by nutrient deprivation.

The strain *Golenkinia* aff. *brevispicula* FAUBA-3 is not European. It was isolated in Argentina [224]. However, there is one European strain, *Golenkinia* aff. *brevispicula* SAG 4.81 [225] isolated from a pond near Dortmund (Germany) by Jeeji-Bai in 1980 with untapped potential for carotenoid production. It was deposited to SAG. According to AlgaeBase, *Golenkinia brevispicula* was mentioned in The Netherlands [30] (Table 2).

### 4.19. Euglena Ehrenberg (Euglenophyceae, Euglenales)

*Euglena* is phylogenetically far from most algae accumulating secondary carotenoids. Chlorophytes and euglenophytes are related to different supergroups of the Eukaryotic Tree of Life, Diaphoretickes and Discoba, respectively [226]. Some *Euglena* members accumulate secondary carotenoids. Although the technology of industrial culturing of them for carotenoid production has not been developed, *Euglena* spp. are promising as a source of these pigments [227,228]. Due to the high diversity of euglenoid metabolic pathways they are also proposed as a source of other valuable compounds [227]. *Euglena rubida* Mainx and *Euglena sanguinea* Ehrenberg (Figure 4s) are the most referenced within the context of carotenoid accumulation. Some of them cause red blooms of freshwater ponds [97,229,230]. Most abundant carotenoid of *Euglena rubida* is astaxanthin (68.5% of total carotenoid amount), another major pigment is mutatoxanthin with minor fractions of others [231] (Table 1). *Euglena sanguinea* predominantly accumulates predominantly astaxanthin (up to 75%) (Table 1). It also contains detectable amounts of the esters of astaxanthin precursors, adonixanthin and adonirubin [229]. Adonixanthin content is also significant, i.e., 13% [229]. Based on the published data, astaxanthin is deposited mainly in the form of fatty acid diesters [229,231]. Some strains of euglenophytes produce the ichthyotoxin euglenophycin, which causes fish mortalities in freshwater aquaculture systems [232]. Certain strains of *Euglena sanguinea* are characterized by the highest euglenophycin content [233]. In *Euglena rubra* it has not been detected [233]. Potential toxicity of *Euglena* strains limits their use as a source of carotenoids, especially in aquaculture.

The Astaxanthin-accumulating strain of *Euglena rubida* with characterized carotenoid profile was isolated from a pool in the grounds of the Branicki Palace, Białystok (Poland) [231]. *Euglena sanguinea* with reported carotenogenesis was collected from a neuston in a eutrophic, nitrogen-poor pond in Eichenbirkig, Fränkische Schweiz, Bayern (Germany) [230] (Table 2). The data on worldwide distribution of *Euglena sanguinea* are summarized by Grung and Liaaen-Jensen [229]. It is distributed in Belarus, United Kingdom, Bulgaria, Czechia, Estonia, Germany, Hungary, Italy, Latvia, Moldova, The Netherlands, Poland, Romania, Slovakia, Spain, and Ukraine (Table 2). Red-colored bloomed water containing *Euglena sanguinea* was sampled from a farm pond in Spydeberg, Ehrenberg, the county of Østfold (Norway) [229]. The strains of carotenogenic euglenoids were deposited to culture collections. The strain *Euglena sanguinea* SAG 1224-30 was isolated from a pool near Debden (United Kingdom) by Pringsheim in 1945 and deposited to SAG [116] (Table 2). The toxic strain *Euglena sanguina* UTEX LB 3117 was isolated in Denmark by Benet in 2011 and deposited to UTEX (Table 2).

### 4.20. Trachelomonas Ehrenberg (Euglenophyceae, Euglenales)

*Trachelomonas* (Figure 4t) is another euglenoid alga able to accumulate secondary carotenoids. In 1963 Green [234] noted accumulation of astaxanthin in addition to common algal carotenoids by *Trachelomonas volvocina* (Ehrenberg) Ehrenberg from a small pond in the Botany Garden at Bedford College (United Kingdom) (Table 1). However, details on its fraction and yield were not provided.

The alga causes red blooms in small water ponds and seas [234]. For example, it caused water blooming of ponds in the United Kingdom and Poland [234,235,236]. It was mentioned in Ireland, Bulgaria, Czechia, Germany, The Netherlands, Poland, Romania, the European part of Russia, Scandinavia, Slovakia, Spain, Sweden, and Ukraine [30] (Table 2). 

### 4.21. Tovellia Moestrup, Lindberg and Daugberg (Dinophyceae, Gonyaulacales)

The genus *Toviella* is related to Dinophyta (supergroup SAR) [226]. *Tovellia rubescens* Pandeirada, Craveiro, Daugbjerg, Moestrup and Calado (Figure 4u) accumulates astaxanthin predominantly in the form of monoesters with fatty acids [99]. Secondary carotenoids are localized in oil bodies in the epicone [99]. Carotenoid accumulation is enhanced by N and P starvation [99]. *Tovellia sanguinea* Moestrup, Gert Hansen, Daugbjerg, G.Flaim and d’Andrea also accumulates a significant fraction of diesters. Notably, its most abundant monoester is with the long-chain polyunsaturated C22:6 microalgae [237]. Carotenogenic *Tovellia* also contains adonirubin and astacene (the form of astaxanthin oxidation) [99,237] (Table 1).

Type locality of *Tovellia rubescens* is the freshwater lake in Gafanha da Boavista, Ílhavo, Portugal [99]. There are no other data on its distribution. *Tovellia sanguinea* was noted in the Lake Tovel [237,238] and other lakes of Trentino Province (Italy) [237,238,239,240] (Table 2), where it causes red blooming.

### 4.22. Diacronema Prauser (Pavlovales)

*Diacronema vlkianum* Prauser (Pavlovales, Haptophyta) is the alga accumulating secondary carotenoids at the stationary growth stage (Figure 4v). Maximal carotenoid content is c.a. 0.6–0.80% of cell dry mass [241,242]. Its main secondary carotenoid is astaxanthin [242] (Table 1). The cells have oil bodies in the cytoplasm [243]. Since the alga also accumulates high amounts of fatty acid residues, it seems that the carotenoid is deposited in the form of fatty acid esters [241,242].

The European carotenogenic strain *Diacronema vlkianum* used in the work by Durmaz et al. [242] was collected on the Portuguese coast and deposited in the AQ/INIAP (Portugal) collection. The strain RCC1546 was collected in the English Channel (France). The strain CCAP 914/1 has been obtained from the sea water Ryde, Isle of Wight, England (United Kingdom). It was also found in Kühnhausen near Erfurt (type locality) [30]. It was mentioned in Ireland, Portugal, Romania and Spain [30] (Table 2).

### 4.23. Rhexinema Geitler (Ulvophyceae, Helicodictyaceae) 

Accumulation of secondary carotenoids was studied in *Rhexinema sarcinoideum* (Groover and Bold) Darienko and Pröschold (Chlorophyta) (formerly *Pleurastrum sarcinoideum* Groover and Bold [244]) by Kopeckýv et al. [133]. Under stress conditions (high light and N-starvation), it accumulates mainly diesters of astaxanthin (Table 1). Detectable amounts of astaxanthin monoesters and canthaxanthin were also observed.

*Rhexinema sarcinoideum* is a freshwater filamentous green alga (Figure 4w). It was isolated from the experimental fields of the Institute of Soil Science and Plant Cultivatio near Puławy (Poland) [101] and from field soil covered with 20 cm snow layer, Chelčice, South Bohemia (Czechia) [245]. The alga was also mentioned in Ukraine and Russia [30] (Table 2).

### 4.24. Trentepohlia Martius (Ulvophyceae, Trentepohliales)

Although the form of *Trentepohlia* (Figure 4x) is not unicellular, it will be also briefly considered here. Representatives of the filamentous *Trentepohlia* genus form bright orange crust on the surface of stones, tree bark, soil, and buildings [246,247]. Although the ability to accumulate carotenoids in these organisms is well-known, the number of works proposing it for biotechnological pigment production is not high (compared with, e.g., *Haematococcus*, *Dunaliella* and *Chromochloris*), e.g., [248,249] mostly due to low growth rate [249]. Predominant carotenoid of *Trentepohlia* spp. is β-carotene with an admixture of α-carotene [248,249,250] (Table 1). Total carotenoid content in these algae can reach 13% of cell dry mass [251].

*Trentepohlia* seems to be ubiquitous in Europe [30]. Diversity of free-living *Trentepohlia* in crusts were studied in Ireland [246]. Here, just several examples on its distribution will be considered. *Trentepohlia abietina* (Flotow) Hansgirg, *Trentepohlia aurea* (L.) Martius, *Trentepohlia* cf. *umbrina* (Kützing) were found. They grew on tree bark, walls of buildings, cement and asbestos sheeting, concrete, and cement walls. In that study, algae were isolated and studied in laboratory cultures. *Trentepohlia* diversity was studied in France. Members of this genus were found on building walls in northern and central France [247]. Distribution of the microalga was studied in the French Alps [72]. *Trentepohlia* spp. inhabiting granite outcrops of river valleys were studied in three climatic zones of Ukraine [252].

### 4.25. Other Microalgae

In some reports the microalgae with unclear taxonomic affiliation are described. Particularly, they are related to *Scenedesmus* spp. and *Chlorella* spp. [8,28,64,76,152,253]. Most representatives were reclassified several times. In many cases, there is no clear information, including genetic data. Moreover, members of these genera also include many non-carotenogenic strains; thus, it is difficult to evaluate the genetic distribution of the representatives accumulating secondary carotenoids. Hence, these representatives are not considered in the current review. 

## 5. Summary of Geographical Distribution

**Figure 5:**
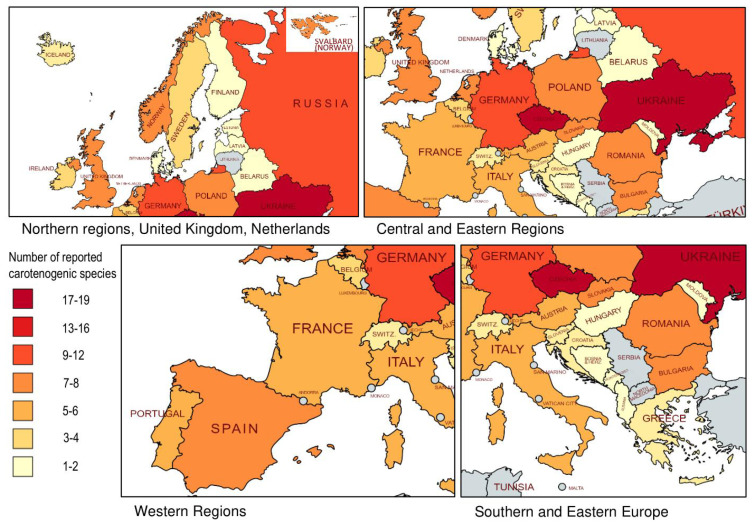
Distribution of reported species of unicellular carotenogenic algae among countries in different regions of Europe (*Trentepohlia* spp., *Scenedesmus* spp. and *Chlorella* spp. were not taken into account). Switz.—Switzerland, Licht.—Lichtenstein, Bosnia & Herz.—Bosnia and Hercegovina. Generated on https://www.mapchart.net (accessed on 20 December 2022).

Unicellular carotenogenic algae are widely distributed throughout Europe (Figure 5). They include most of reported algae able to accumulate secondary carotenoids: *Haematococcus*, *Ettlia*, *Dunaliella*, *Chromochloris*, *Chloromonas*, *Chlainomonas*, *Sanguina*, *Coelastrella*, *Bracteacoccus*, *Halochlorella*, *Tetraëdron*, *Deasonia*, *Chlorosarcinopsis*, *Acetabularia*, *Pseudospongiococcum*, *Protosiphon*, *Botryococcus*, *Golenkinia*, *Trachelomonas*, *Toviella*, *Diacronema, Rhexinema*, and *Euglena*.

The highest number of registered species was found in Ukraine, Czechia, Germany, and Russia. Relatively high number was also observed in Spain, Romania, Slovenia, Bulgaria, The Netherlands, and Norway as well as in Austria, Italy, France, Poland, and the United Kingdom (Figure 5). Notably, the high number of reported species can be explained by the presence of large biotopes with adverse conditions. Indeed, mountain ranges, such as Alps in Italy and Austria, Sierra Nevada in Spain, High Tatra in Slovakia and Poland, and Giant Mountains in Czechia, Vitosha, Central Balkan Mountains, and Pirin Mountains in Bulgaria. High diversity of snow alpine microalga was recorded there [51,73,86,107,143,144,145,146,147,148,149,151,152,153,154,155,156,157,158,159,160]. The same is true for polar snow valleys and mountains of Norway, especially on the Svalbard Archipelago [73,86,120,145,150,161]. Carotenogenic algae are also abundant in the Polar zone of the White Sea coast with temporary dried small rock ponds with semi-saline water [87,88,113]. Studies of ponds with extreme salinity also have contributed to the number of registered carotenogenic species. These are the cases of, e.g., Monzon (Spain), Lacul Sărat (Romania) and Elton (Russia) [125,126,128]. There are many extreme biotopes in Ukraine, which can explain the high number of strains there. They include hypersaline ponds in Kherson Oblast, Odesa Oblast, and Crimea, as well as the Carpathian and Crimean Mountains [37,129,130,131,185]. Another possible explanation of distribution of reported species of carotenogenic microalgae is traditions of scientific groups studying these objects. The best examples are studies of snow algae in European alpine regions by the same authors and studies of the diversity of carotenogenic algae in Ukraine, where it was proposed for the first time to use halophilic algae as a source of carotenoids. The studies on diversity of soil and aeroterrestrial species in Germany and the United Kingdom also have promoted generation of knowledge in the field of carotenogenic algae diversity.

## 6. Conclusions and Perspectives

Discussed species of European carotenogenic algae are related to three different clades of the Tree of Life, Diaphoretickes, SAR, and Discoba. Although in some reported strains carotenoid yields are not high, they could be promising objects for biotechnology. They can produce pigments from a wide secondary carotenoid spectrum: β-carotene, astaxanthin, canthaxanthin, echinenone, adonixanthin, and adonirubin. Some species can be used for production of mixtures of valuable carotenoids. This offers new opportunities to create natural products with a combination of benefit properties. In this regard, new studies on optimization of culturing conditions of poorly characterized strains are required. Isolation of the strains from different natural habitats, opens up prospects for culturing in different conditions, especially at low temperature or in seawater. Collectively, the existing data on the diversity of carotenogenic algae is valuable for further research on their unrevealed biotechnological potential.

## Data Availability

Not applicable.
